
JOURNAL INFORMATION
==============================
NLM Title Abbreviation: Behav Neurol
Journal ID: Behav Neurol
NLM Journal ID: 8914585
Journal ID: BN

Behavioural Neurology

ISSN: 0953-4180
EISSN: 1875-8584
Publisher: Wiley

ARTICLE INFORMATION
==============================
PMCID: PMC5215432
PMID: 28337050
DOI: 10.3233/BEN-2012-129001
Article ID: 794524
Article version: 1
Subjects: Guest Editorial

Neuropsychology across the Lifespan: Proceedings of the Third Meeting of the Federation of European Societies of Neuropsychology

Ptak Radek 1 2 *
Vingerhoets Guy 3
1 Division of Neurorehabilitation Department of Clinical Neurosciences Geneva University Hospitals Geneva Switzerland
2 Medical School University of Geneva Geneva Switzerland
3 Department of Experimental Psychology Ghent University Ghent Belgium
*Radek Ptak: radek.ptak@hcuge.ch
Print publication date: 2013
Volume: 26
Issue: 3
First page: 165
Last page: 165
Received 2012 May 21; Accepted 2012 May 21
Copyright: Copyright © 2013 Hindawi Publishing Corporation and the authors.
Copyright year: 2013
License: This is an open access article distributed under the Creative Commons Attribution License, which permits unrestricted use, distribution, and reproduction in any medium, provided the original work is properly cited.
License URL: https://creativecommons.org/licenses/by/3.0/

==============================
Behavioural Neurology 26 (2013) 165
DOI 10.3233/BEN-2012-129001
IOS Press

### Guest Editorial

### Neuropsychology across the Lifespan: Proceedings of the Third Meeting of the Federation of European Societies of Neuropsychology

Radek Ptak[a,b,*] and Guy Vingerhoets[c]
[a]*Division of Neurorehabilitation, Department of Clinical Neurosciences, Geneva University Hospitals, Geneva, Switzerland*
[b]*Medical School, University of Geneva, Geneva, Switzerland*
[c]*Department of Experimental Psychology, Ghent University, Ghent, Belgium*

The *Third Meeting of the Federation of European Societies of Neuropsychology* (ESN) took place at the Congress Centre in Basel, Switzerland, from 7–9 September 2011. The theme of this meeting, which assembled more than 400 clinicians and researchers from many European countries as well as from North America, Asia and Australia, was 'Neuropsychology across the Lifespan'. This theme gave the opportunity to discuss a large range of current neuropsychological topics pertaining to different developmental stages and age groups. We are grateful to the Editors of *Behavioural Neurology*, Argye Hillis-Trupe and Stefano Cappa, to give us the opportunity to present here a selection of individual contributions in a condensed format. The studies described here were included as oral presentations in one of four sessions: Reading and Alexia (Starrfelt et al., Auclair-Ouellet et al.), Space and Action (van der Ham et al., Bonato et al.), Plasticity and Rehabilitation (Sedda et al., Dubovik et al., Gutiérrez Iglesias et al., Duits et al.), and Memory and Amnesia (van Geldorp et al., Marques, Barban et al., Jonin et al., El Haj et al.). We hope these excellent contributions motivate you to participate to one of the future meetings of the ESN.

*Corresponding author: Radek Ptak, Division of Neurorehabilitation, Geneva University Hospitals and Faculty of Medicine, University of Geneva, 26, Av. de Beau-Séjour, 1211 Geneva 14, Switzerland. Tel.: +41 22 382 35 24; Fax: +41 22 382 83 38; E-mail: radek.ptak@hcuge.ch.

ISSN 0953-4180/13/$27.50 © 2013 – IOS Press and the authors. All rights reserved
